# A community-integrated home based depression intervention for older African Americans: descripton of the Beat the Blues randomized trial and intervention costs

**DOI:** 10.1186/1471-2318-12-4

**Published:** 2012-02-10

**Authors:** Laura N Gitlin, Lynn Fields Harris, Megan McCoy, Nancy L Chernett, Eric Jutkowitz, Laura T Pizzi

**Affiliations:** 1 Johns Hopkins University, 525 Wolf Street, Baltimore, Maryland 21205, United States of America; 2 Center in the Park, 5818 Germantown Avenue, Philadelphia, PA 19144, United States of America; 3 Thomas Jefferson University, 1015 Walnut Street, Suite 115, Philadelphia, PA 19107, United States of America; 4 University of Minnesota, Health Policy and Management, 420 Delaware Street SE, MMC 729Minneapolis, MN 55455. United States of America

## Abstract

**Background:**

Primary care is the principle setting for depression treatment; yet many older African Americans in the United States fail to report depressive symptoms or receive the recommended standard of care. Older African Americans are at high risk for depression due to elevated rates of chronic illness, disability and socioeconomic distress. There is an urgent need to develop and test new depression treatments that resonate with minority populations that are hard-to-reach and underserved and to evaluate their cost and cost-effectiveness.

**Methods/Design:**

Beat the Blues (BTB) is a single-blind parallel randomized trial to assess efficacy of a non-pharmacological intervention to reduce depressive symptoms and improve quality of life in 208 African Americans 55+ years old. It involves a collaboration with a senior center whose care management staff screen for depressive symptoms (telephone or in-person) using the Patient Health Questionnaire (PHQ-9). Individuals screened positive (PHQ-9 ≥ 5) on two separate occasions over 2 weeks are referred to local mental health resources and BTB. Interested and eligible participants who consent receive a baseline home interview and then are randomly assigned to receive BTB immediately or 4 months later (wait-list control). All participants are interviewed at 4 (main study endpoint) and 8 months at home by assessors masked to study assignment. Licensed senior center social workers trained in BTB meet with participants at home for up to 10 sessions over 4 months to assess care needs, make referrals/linkages, provide depression education, instruct in stress reduction techniques, and use behavioral activation to identify goals and steps to achieve them. Key outcomes include reduced depressive symptoms (primary), reduced anxiety and functional disability, improved quality of life, and enhanced depression knowledge and behavioral activation (secondary). Fidelity is enhanced through procedure manuals and staff training and monitored by face-to-face supervision and review of taped sessions. Cost and cost effectiveness is being evaluated.

**Discussion:**

BTB is designed to bridge gaps in mental health service access and treatments for older African Americans. Treatment components are tailored to specific care needs, depression knowledge, preference for stress reduction techniques, and personal activity goals. Total costs are $584.64/4 months; or $146.16 per participant/per month.

**Trial Registration:**

ClinicalTrials.gov #NCT00511680

## Background

Depression, a common, debilitating but treatable condition among older adults, contributes to poor quality of life and functional decline and is one of the most serious complications of chronic conditions [1]. Depression worsens health conditions and exacerbates disability contributing to a pernicious downward spiral [2]. Even mild to moderate depressive symptoms are associated with difficulties performing daily activities and may amplify and prolong inflammatory responses after infection [3].

Depression prevalence ranges from 15 to 17%, but rates are twice as high among individuals with physical health problems, functional disabilities, low socioeconomic status and receiving home care or related services [4]. Older African Americans in the United States (USA) are at particular risk due to high rates of chronic illnesses associated with depression (diabetes, heart disease), and social, economic and environmental detriments. Evidence also suggests higher prevalence of depressive symptoms for this group than previously reported [5]. The African American Health Study of 998 community-dwelling African Americans found 21.1% with clinically relevant depressive symptomatology [6]. Of 150 older poor African Americans attending outpatient rehabilitation, 30% scored positively for depression [7]. A survey of 156 African American senior center members found 24.2% with mild to moderate severe depressive symptoms [8].

In the USA, most older adults are screened for depression and treated in primary care settings, yet frequently do not receive the recommended standard for depression care. A consistent finding is that older African Americans tend to be underdiagnosed in primary care and underutilize mental health services [9,10,11)]. Moreover, with few exceptions, mental health treatments have not been developed or evaluated specifically for older African Americans [12]. However, when depression treatments including medication and/or cognitive therapies are provided, outcomes for this group are beneficial and long-lasting [13].

Previously tested system-level depression studies target primary care settings. These interventions principally involve collaborative care management treatments with large trials reporting impressive treatment effects [14-17]. However, these models have not been developed specifically to outreach to older African American communities and, with few exceptions outcomes have not been evaluated for this group [12]. Also, these models are difficult to sustain due to limitations in clinical and reimbursement structures unsupportive of care coordination activities essential to their success.

Moreover, studies consistently show that African Americans continue to obtain poorer quality of depression care than White patients [9,10,18,19]. Barriers to depression care persist at the patient level and include stigma and lack of depression knowledge [11,20]; provider level due to lack of knowledge and training in assessment and treatments [9], and systems level due to lack of care coordination and sustainable infrastructures [21]. Persistence of mental health disparities for older African Americans in the USA, higher depression rates than previously found, coupled with continued unequal access to culturally relevant mental health treatments, point to the need to advance more powerful care models and interventions for this group. Developing and testing new service approaches that improve access to depression detection, referral and treatment tailored to treatment preferences of older African Americans remains an important public health priority [22].

An overlooked and underutilized system of care for depression detection and treatment is the aging network of services, a federally and state-funded system in the USA providing an array of social services to over 9 million older adults, many of whom are vulnerable and underserved [23,24]. One promising depression program building upon previously tested approaches and offered through the aging network, Healthy IDEAS (HIDEAS; Identifying Depression, Empowering Activities for Seniors), has shown modest positive client outcomes including enhanced depression knowledge, decline in symptomatology, and clinician adoption [25,26]. HIDEAS involves systematic screening, depression education, referral and linkage and engaging clients in behavioral activation by case managers. Nevertheless, HIDEAS was not tested in a randomized trial and less than half (44.7%) of the 94 participants received behavioral activation, with only a small percentage being African American [26].

A few studies evaluating intervention delivery at home suggest that older adults prefer home over clinic-based treatment [8], and home sessions may lead to better treatment acceptance [16], fewer nursing home admissions and in-patient care days, as well as be cost effective [27].

We designed Beat the Blues (BTB) to be delivered through a community-based organization such as senior centers. Senior centers routinely assess older adults for service needs and health status, serve as initial contact for a continuum of aging services, and provide a safety net offering meals, health checks, care management and referral services. Individuals reluctant to consult primary care physicians or mental health specialists may be more comfortable disclosing depressive symptoms to skilled senior center staff with whom they have rapport. Although senior centers potentially reach large numbers of vulnerable older adults at risk for behavioral health problems, most do not systematically screen for depressive symptoms or offer evidence-based depression treatments. Building senior center capacity to detect depressive symptoms and deliver effective non-pharmacological treatments may compliment other care systems and optimize mental health programming for underserved.

BTB builds upon the successes and lessons learned from prior depression treatment efforts but it also differs from previous efforts in important ways. First, delivery is integrated with staffing and daily routines of a community-based agency, a senior center versus a mental health clinic or primary care setting, enhancing potential for normalization and sustainability in that setting. Second, BTB is culturally sensitive; its name reflects the characterization of depression by the target group; and treatment components resonate with their preferred coping approaches. As activity is a primary strategy for coping with adversity among older African Americans [28], behavioral activation was chosen as an essential component of BTB. This approach, shown to be effective in previous depression trials, helps participants reengage in self-identified meaningful activities. Third, while each treatment component of BTB has been shown to be effective in other trials, they have not been combined into one program and systematically tested with older African Americans.

BTB is currently being formally evaluated in a prospective parallel two-group randomized trial. The study is designed as a practical trial embedded in a senior center. This article describes trial procedures, the intervention, enrollment, and program costs. Trial results and cost effectiveness will be reported in future articles.

## Methods/Design

The BTB study protocol received ethical approval from the Institutional Review Board at Thomas Jefferson University (Control #06F.551) in which the protocol was initiated; with ethical approval also subsequently received by the Institutional Review Board at Johns Hopkins University (Control # NA-00046775) upon appointment of the Principal Investigator and lead author. Also, a Federal Wide Assurance Agreement between the senior center agency (Center in the Park) in which the study was implemented and Thomas Jefferson University was obtained.

BTB reflects a partnership between a university research center and a senior center with each contributing to the plan and execution of the study and model design while preserving internal validity and control of a randomized trial. Agency staff are trained in human subjects and participate in recruitment efforts, conduct depression screenings, and deliver the home intervention; research staff provide oversight of trial procedures, jointly participate in recruitment efforts, train agency staff in depression screening, implement randomization procedures, and provide oversight of intervention delivery and monitoring of treatment fidelity. The design optimizes experimental control while building capacity of the participating senior center to screen and deliver BTB; hence, maximizing potential for sustainability and self-sufficiency at trial conclusion if BTB is efficacious and cost-effective. Additionally, the partnership enables the senior center to access mental health researchers and experts to provide oversight and guide implementation. The academic institution maximizes recruitment and trial success through involvement upfront of end users of the intervention. This approach may also enhance sustainability if the intervention is shown to be effective.

General procedures include two sequential depression screenings of individuals by senior center staff over two weeks using the Patient Health Questionnaire (PHQ-9). Those eligible (PHQ-9 ≥ 5) and willing provide written consent using an approved Institutional Review Board (IRB) form (Thomas Jefferson University IRB control #06f.551) and receive a baseline home interview conducted by academic partners. Participants are then randomized to receive BTB immediately or 4 months later (wait-list control). All participants are reassessed at 4 and 8 months by academic partners who remain masked to study assignment. The design allows for a true two-group comparison at 4 months to evaluate intervention effects on symptom severity (primary end point), quality of life indicators, anxiety, depression knowledge, and functional disability (secondary endpoints); an evaluation of long-term (8 months) effects for the initial treatment group; and whether wait-list controls derive similar benefits from 4 to 8 months. Treatment fidelity is enhanced through use of treatment manuals, session implementation checklists, audiotaping and rating of randomly selected sessions, one-on-one supervision and bi-weekly group debriefing sessions involving structured case presentations and review of treatment documentation.

### Aims and study hypotheses

Our primary aim is to test the immediate effect of the intervention at 4-months on symptom severity (PHQ-9 severity score) in African American older adults (primary trial outcome; between group comparison). Our hypothesis is that participants in the initial treatment group will report fewer depressive symptoms in comparison to control group participants receiving usual care. We also seek to test the maintenance effect of the intervention at 8-months on depression (within group comparison). Our hypothesis is that participants in the initial treatment group will maintain reduced symptom presentation from 4 to 8 months and the wait-list control group will show similar reductions in depressive symptoms as the initial treatment group (within group comparison).

A secondary aim is to evaluate whether BTB has immediate (4 months) and long-term (8 months) effects on quality of life indicators, functional difficulty, anxiety, and depression education and behavioral activation levels. We expect that the initial treatment group will show improvements in these areas relative to the wait-list control group, that improvements will be maintained at 8 months and that the wait-list control group will demonstrate similar improvements at 8 months following their receipt of the intervention.

Several exploratory aims are also proposed. We will evaluate the mechanism of action, or pathways, by which treatment gains are obtained. Given that behavioral activation represents conceptually the key active ingredient of the proposed intervention, we plan to evaluate its mediational effect. We will also evaluate whether the intervention has a differential treatment effect based on a study participant's gender, age, and living arrangement (alone or with others). Given that previous research suggests that participant characteristics may moderate depressive symptoms and treatment outcomes, these exploratory analyses will provide insight as to whether this particular treatment benefits some groups more than others.

### Recruitment, eligibility and randomization

Participants enrolled in the trial were recruited from two sources, a program providing short-term in-home support for individuals who are medically comprised (referred to as the In-Home Support Program; IHSP) or the community at-large. IHSP provides homebound elders assistance regaining independence after illness or hospitalization through home health and homemaker services, home delivered meals, transportation, or companions but does not typically involve formal and systematic depression screening. Recruitment from IHSP involved integrating depression screening in the assessment process of participants in the program. Recruitment to the community at-large involved print media, presentations to community groups, and print media.

Eligibility criteria for BTB include being African American; ≥ 55 years of age; English speaking; cognitively intact (MMSE > 24), and a score ≥ 5 on the PHQ-9 on two sequential testing occasions. Other criteria are practical and include having a home telephone and planning to live in the area for 8 months. Individuals are not eligible with serious mental illness histories, life-limiting illnesses, involvement in another clinical depression trial, or if living in assisted living or nursing home facilities. Individuals receiving anti-depressant medications or other mental health treatments are eligible.

As individuals from IHSP may be more medically and functionally compromised than those enrolled from the community at-large, we used a stratified randomized design to assure equivalence between groups along this dimension. Permuted block randomization with each stratum (IHSP versus other recruitment source) to control for possible changes over time in subject mix was employed. The blocking number was developed by the trial senior statistician and was not disclosed to the investigative team.

### Measures

In selecting measures for this study, we chose those with known reliability and validity; sensitivity to change that is expected to occur as a consequence of intervention; relevance to and wide usage in depression research, and acceptability by the target population. Our primary outcome measure is the Patient Health Questionnaire (PHQ-9), a brief, 9-item self-report measure specifically developed as an assessment tool for the diagnosis of depression in primary care. In addition to its psychometric soundness and demonstrated reliability, validity, sensitivity and specificity, the measure is also widely used in community-based settings and research trials. It yields a depression severity score (0 to 27) and a diagnostic category (minimal to none, mild, moderate, moderate severe, severe) mapping on to the 9 DSM-IV. Administration involves initially asking two items (loss of interest; feeling upset, distressed or depressed). Individuals reporting that either symptom occurs more than half the time over past two weeks are administered 7 other items [29]. Thus, two scores can be derived: symptom severity and categorical diagnosis. We also consider as a complementary secondary outcome a change in categorical diagnoses in which we seek to determine the number of persons who shift to none to minimal diagnostic category at 4 months. A categorical approach provides a clinical interpretation of treatment efficacy and also whether the intervention addresses certain syndromes better than others (e.g., minor depressive disorder may be more responsive to the proposed intervention than major severe depression). While the PHQ-9 is a self-report measure, self-report has been shown to be highly associated with clinical determinations. Also, the measure has been shown to be effective when administered in person or via telephone. The secondary outcome measures for the trial are presented in Table 1.

**Table 1:** Secondary Outcome Measures for BTB Trial

Domain	Description of Measure	# of Items
Functional difficulties	Level of difficulty (1 no difficulty to 5 unable to do due to health problem) with items reflecting ambulation, self-care and instrumental activities of daily living [31].	18
Depression knowledge	Self-efficacy concerning ability to recognize and treat depression rated from absolutely confident (4) to not confident at all (1) [32].	10
Behavioral Activation	Behavioral Activation Scale (Abbreviated) contains items rated on a scale from 0 not at all to 6 completely and address activation in a range of daily activities (e.g., stayed in bed too long; I accomplished the goals I set out to do to) [33].	26
Anxiety	State Anxiety Scale in which items are endorsed as 1 (very much) to 4 (not at all) [34].	10
Quality of daily life	Adapted from the Perceived Change Index, items reflect mood, sleep quality and daily well-being rated from 1 (gotten much worse in past month) to stayed the same (3) to improved a lot (5) [35].	14
Overall quality of life	Adapted from the Quality of life for Alzheimer's Disease items are assessed from 1 (poor) to 4 (excellent) concerning experience of pleasure, hopefulness, positive relationships with family and friends [36].	12

### BTB Intervention

Up to 10 one hour sessions occur over 4 months. A home visit can be replaced with check-in telephone calls once steady progress is demonstrated. Sessions integrate five treatment components previously shown to be effective and modified in BTB to resonate with cultural preferences: case management, referral and linkage, depression education, stress reduction techniques, and behavioral activation. (Table 2) Critical is tailoring to participants' depression knowledge level, care management needs, and self-identified goals and behavioral activation plans.

**Table 2:** Intervention Component, Content, and Modification for Target Population

Component	Content	Modifications for Target Population
Education [14,25]	Education provides foundation for introducing other elements of the intervention. It enhances readiness of individuals to address emotion-laden concerns. Specifically, education was provided on: 1) depressive symptoms, 2) how to talk to doctor about symptoms; and 3) relationship of depression to activity and negative cycle of disengagement	1) Used participant's words and own labeling to describe feelings (e.g., "I am feeling blue"). 2) With rapport and only over time, feelings are then labeled as symptoms of "depression." 3) Discussed how to talk to a doctor of a different race. 4) Withdrawal of activity related to specific activities person identified as valued. 5) Used visual mood rating scales and calendars with big print to minimize vision and literacy challenges.
Care management [12,14]	Care management has been found to be more effective than therapy or medication alone for low income elders for whom financial, functional disability and lack of social resources may contribute to depressive symptoms. Specifically this involved: 1) assessment 2) coordination with other services/care management; 3) problem identification and resolution	1) Considered a wide range of care needs most relevant to this population including home repairs, financial concerns, home and neighborhood safety, family conflict. 2) Housing and neighborhood difficulties were evaluated to determine whether person could remain in setting.
Referral and linkage [14,25]	Referrals and linkages are derived from the care management assessment and may include: 1) physician referral for medication review and management; 2) link to psychiatric/psychological follow-up; 3) link to physician for chronic disease management; 4) referral and linkage to other services (e.g., home repairs, financial or legal advisors)	1) Referrals made to vetted community-based service providers sensitive to participants' resources and cultural preferences. 2) For individuals identified as in need of more mental health support, helped make bridge to these services
Stress reduction	Provides immediate, easy-to-learn technique to reduce stress of person and introduce relationship of action and mood change. Specific techniques included: 1) deep breathing; 2) counting; 3) use of music of personal interest	1) Recognition of importance of spirituality and possible objection to meditation as a stress reduction activity. 2) Use of relaxation CD/Tape with participants who did not want to participate in deep breathing exercise.
Behavioral Activation [14,16,25]	Approach is designed to increase frequency of pleasant events and provide positive reinforcement. Self-identification of goals/action plan promotes activation. The approach involves: 1) identification of valued activities and goals; 2) establishment of plan of action for goal attainment; 3) monitoring and adjustment of plan/goals; and 4) identification of new goals and steps to attain them and reinforcement and validation.	1) Awareness and identification of appropriate community-based programs and services that could help participants link to meaningful activities. 2) Use of "Pro/Con" lists and motivational interviewing in formulation of feasible goals. 3) Link individual to senior center to enable continuation of activity participation in a planful, structured setting.

Initial sessions occur weekly and then bi-weekly. In session one, building rapport and a therapeutic relationship begins; also, interventionists assess care management needs and begin education about depressive symptoms making connections between behavior and mood. Explained is that with depression, activities once pleasurable are often stopped which may heighten symptomatology. Likewise, previously pleasurable activities may no longer be pleasurable. Interventionists also discuss sharing depressive symptoms with physicians (if medication may be necessary or for future consideration) and strategies to use when physicians are of a different race. Interventionists introduce deep breathing as a basic stress reduction technique and help participants identify stressful points in the day for its use. In sessions 2-3, interventionists engage participants in simple problem solving to resolve identified care management needs (medical, housing repairs, relocation needs, social, benefits/entitlements), and works on a care management plan involving coordination, referral and linkages to other services if necessary. Other stress reduction techniques (counting, music) are introduced to provide additional tools. In sessions 4-5, interventionists continue addressing care management needs and introduce behavioral activation. Interventionists review daily routines and help participants select a goal and specific activity to add pleasure and personal satisfaction. Active problem solving and motivational interviewing techniques help participants achieve identified activity goals. Potential barriers in carrying out activities are identified and solutions derived which may also require care management (arranging transportation) to engage in desired activities. Sessions 6-8 involve reinforcement of activity engagement, identification of new activity goals and specific steps to achieve them. In sessions 9-10, interventionists review and reinforce all techniques, help participants identify and use behavioral activation strategies, and obtain closure. Approximately 6-7 sessions are devoted to behavioral activation.

### Wait-list control group

The control group does not receive any study-related contact following the baseline interview. After completion of the 4-month reassessment, participants in this group receive the entire BTB intervention as delivered to the initial treatment group.

### Analytic plan

We based our sample size calculation on the following assumptions: a) one primary outcome (severity of depressive symptoms); b) treatment effect sizes reported in previously published intervention studies testing one or more components of the intervention we employ in BTB (effect sizes range from .70 at 6 months and .35 at 12 months in one trial^16 ^to .40 at 3 and 6 months and .60 at 12 months for another trial^14^); c) ability to detect a medium effect size of Cohen's d = 0.40; and d) type I error rate of .05 for the single primary hypothesis. A larger effect size may not be plausible, whereas a smaller effect size brings us at or near levels where the study could have statistical but not clinical significance. In order for this brief, targeted intervention to be worth implementing in a service context, it must yield more than trivial effects. Since our planned analyses are primarily analysis of covariance, we calculated the power of a two-sample t-test. Because we will use analysis of covariance to adjust for baseline values, we expect to have adequate precision and power. To attain 90% power for a two-sided alternative hypothesis using a t-test to compare the two treatment groups with respect to 4-month values require 172 study participants for an effect size of .40. We planned to recruit an additional 20 participants for a total of 192 participants (96 per group). The additional study participants allowed for a conservative estimated attrition rate of 10%. To obtain the necessary sample size of 172, we needed to enroll 192 study participants. The statistical power for secondary analyses is the same as for the primary; 90% for a 0.40 effect size with 5% two-tailed tests. Although we did not conduct power calculations for exploratory aims, these analyses are important in order to refine research questions and hypotheses worthy of future consideration, refinement of the intervention and its dissemination.

The focus of the primary analysis is to examine the effect of the intervention on symptom severity based on the principle of "intention to treat." That is, all subjects assigned to the initial treatment group will be part of the primary analyses regardless of the actual number of intervention contacts received. This approach tests the effects of the intervention without accounting for the extent to which study participants actually receive the intervention. It effectively penalizes the intervention if subjects are not willing to receive it or if subjects receive different doses or exposure to treatment. We will calculate adjusted mean differences in treatment effects on the outcome measure at 4-months using analysis of covariance. We will include as covariates the baseline value of the outcome measure and the stratification variable (recruitment source). Other background characteristics may also be used as covariates if large or statistically significant differences between initial treatment and wait-list control group participants are found for those variables in analyses conducted prior to any efficacy analyses. Prior to conducting the ANCOVA, we will test the normality assumption for the dependent measure by examining the distribution of the residuals. If the residual distribution is skewed, we will use a transformation of the data.

For purposes of reporting results, we will also compute proportions improved. We will use two definitions of improvement: any reduction in PHQ-9; a reduction sufficient to move the patient at least one severity category lower. Note that we do not have to worry about the no symptom (0-4) at baseline group in this definition, as that group is not eligible. The comparison of treatment to wait-list control will be with odds ratio and 95% confidence intervals. We will also examine clinical significance by conducting subsidiary analyses that examine the proportion of individuals in treatment compared to controls who change diagnostic categories. Finally, in another set of analyses we will incorporate measures of the extent to which treatment has been received to evaluate relationship of dose/intensity to treatment outcome. This is for reporting purposes and we are not powering for these alternative endpoints.

As to our second aim, we seek to evaluate whether participants in the initial treatment group maintain reduced symptom presentation from 4 to 8 months. We view retention as a one-sided concept such that any beneficial effect of the intervention at four months is improved or at least maintained at 8 months. Lack of retention is then considered a non-trivial loss of benefit. In the language of clinical treatment trials, this is a "clinical equivalence" or "non-inferiority" hypothesis. Thus, our analyses for our second aim will focus on estimating the amount of 4-month benefit that is retained at 8 months for the treatment group only. We will use confidence intervals in reporting results in order to understand how much loss of benefit is consistent with the data we obtain. Given that the main outcome measure, PHQ-9, has cut off points related to DSM IV depression conditions (e.g., dysthymia, major depression), we will be able to evaluate loss of benefit and retention of effect within the treatment group based on clinically relevant features.

For exploratory aims, we seek to evaluate the mediational role of behavioral activation. We will use separate regressions in which the first regression will evaluate the relationship between 4 month values of the behavioral activation scale (the mediator) and symptom severity as measured by the PHQ-9 at 4 months. The second regression will evaluate the relationship between group assignment (the independent predictor variable) and symptom severity at 4-months (PHQ-9, the criterion). The third regression will evaluate the relationship of treatment assignment (predictor) to PHQ-9 severity scores (criterion) after entering behavioral activation values (mediator). To evaluate the strength of the mediation effects, we will compute the ratio of the unstandardized betas for treatment assignment (beta from regression III divided by beta from regression II) with values less than 1.00 indicating potential mediation. We will repeat these analyses using 8-month values of the PHQ-9. Another exploratory aim is to evaluate whether the intervention has a differential treatment effect based on a study participant's gender, age, or living arrangement (alone/with others), factors which have been shown to be differentially related to depression. To evaluate differential treatment effects at 4-months, we will use a similar analytic strategy as for the main effect model. ANCOVAs will be used in which the covariates will be the baseline value and specific participant characteristic (e.g., male/female; live with other or alone; age 60-64 versus 75+). We will then add an interaction term of group assignment by participant characteristic.

### Cost analysis

Economic evaluations of home support programs for depressive symptoms targeting older African Americans and which can be delivered by trained staff of a community-based agency are non-existent. Cost analyses are critical for evaluating the translational and implementation potential of such trials. We have calculated the cost of delivering BTB by senior center staff trained in the program (presented below); and we will calculate the cost effectiveness of BTB from a societal perspective that employs two relevant outcome measures: cost per quality adjusted life year (QALY; Euro-QOL 5D vs. health utility measures (HUI 2 and HUI3), and cost per reduction in depression symptoms (PHQ-9). Cost-effectiveness will be measured as the average incremental difference between cost of BTB and the control group divided by the difference in health utility (measured as QALYS) between treatment and control periods at 4 months, and at 8 months for the subsample of participants who receive BTB for the first 4 months and are observed for another 4 months. Univariate and probabilistic sensitivity analyses will be conducted to determine the incremental cost effectiveness of BTB at 8 months, and will aid in determining the robustness of the model when inpatient and outpatient medical and medication costs are varied. As funds for the cost analyses were obtained after the trial commenced, the cost analyses will involve a subset (n = 130/208) of participants for which we are able to collect QALY measures.

### Recruitment and enrollment outcomes

Screening and enrollment results to date provide an indication of the feasibility of the BTB trial. A total of 17 senior center care managers were trained to use the PHQ-9 to screen for depression. Although screening and enrollment is closed for this trial, the screening protocol has become fully integrated into routine intake and reassessments across all senior center programs including IHSP.

A total of 703 older adults were initially screened over a 2 1/2 year recruitment period; 440 from IHSP; 263 from the community at-large (senior center members and elsewhere). Of 703, 390 (55%) scored positively (PHQ-9 ≥ 5) and were eligible for the second screen. Of 440 from IHSP, 137 (31%) were eligible for the second screen suggesting the importance of detection for this medically compromised population. Of 263 community at-large, 253 (96%) scored positively suggesting that media and outreach efforts successfully targeted individuals with depressive symptoms, individuals correctly self-identified as feeling depressed, and African Americans with depressive symptoms will volunteer for a non-pharmacological trial.

Of the initial 390 positive screens, 279 (72%) were successfully screened a second time of whom 192 (69%) were eligible and willing to participate in BTB. Another 16 individuals with initially negative PHQ-9 scores were screened 3 months later, became eligible, and enrolled in the trial for a total of 208 participants. As these participants contacted the study team prior to meeting the targeted sample size, they were included in the trial.

### Sample characteristics

Participants are on average 69.5 years, mostly single, female, and with ≥ high school education. Over half (67.79%) report some to a lot of difficulty paying for basics, and have an average of 6.6 health conditions with more than a third having diabetes (42.8%), high blood pressure (78.8%), high cholesterol (58.2%), and arthritis (76.4%). Only 19.2% were on an anti-depressant medication and 16.8% on an anti-anxiety medication whereas 52.9% were on pain medications. The average second PHQ-9 score was 13 with 72% reporting moderate to severe symptoms. (Table 3)

**Table 3:** Baseline Characteristics of BTB Sample (N = 208)

Characteristic	Mean (SD)	%
Age^a^	69.5 (8.7)	
Sex		
Male		22.1
Female		77.9
Education		
< HS		21.2
HS/GED		29.3
> HS		49.5
Paying for basics		
Not difficult at all		19.7
Not very difficult		12.5
Somewhat difficult		37.5
Very difficulty		30.3
Marital status		
Single		88.0
Married		12.0
Number of health conditions	6.6 (3.5)	
Antidepressant medication		19.2
Anxiety medication		16.8
Pain medication		52.9
PHQ9 Score (second screen)	13.0 (4.9)	
Minimal/no depression (0-4)		0.0
Mild depression (5-9)		28.5
Moderate depression (10-14)		35.3
Moderate/severe depression (15-19)		24.2
Severe depression (≥ 20)		12.0

^a^N = 207.

### Program costs

The total cost of BTB, reported in 2010 dollars, was obtained using a microcosting approach where total costs represent the sum of microcosts in 3 main categories: screening, intervention delivery, and program supervision. (Table 4) To capture costs, senior center supervisors and interventionists kept detailed logs of time spent conducting BTB tasks. Though double screening over a two week period is required to confirm trial eligibility, costs for the first screening only are reported with the assumption that only one screening would be performed if BTB is translated for real world settings. Interventionists record real time in preparation, documentation and implementation for each session delivered in person or telephonically. Staff training time for interventionists is also captured and converted to costs based on application of hourly wage rates. Supervisors of interventionists at the senior center account for time spent with each interventionist in supervision. Also, cost of managing adverse events during the trial, such as suicidal ideations, is captured.

**Table 4:** Costs for Delivery of Beat the Blues to Treatment Group to Date (n = 122)

	Description	How Measured	Mean Cost Per Participant (SD)
**Screening**			
A. Screening Cost	Screening by a trained senior center care manager staff	Time spent screening potential participants multiplied by screener's wage rate + fringe benefit costs divided by sample size	**$2.63 (1.51)**
Intervention Delivery*			
B. Intervention Delivery Cost Per Person (Cost Per Session X Number of Sessions)	Intervention conducted by a trained senior center staff member	Time conducting intervention multiplied by wage rate of interventionist + fringe benefit costs divided by sample size	**$197.31 (133.00)**
**Participant Contact Outside of Intervention Delivery^+ ^**			
C. Total Cost of Contacts Outside Intervention (Cost Per Contact X Number of Contacts)	All contact outside of the intervention by the interventionist (telephone support, preparation and documentation, mailing, faxing, and other)	Time spent in contact outside of intervention multiplied by wage rate + fringe benefit costs of interventionist divided by sample size	**$84.00 (60.00)**
**Travel**			
Mileage Reimbursement (Miles X $0.55)	Travel to and from homes of study participants by interventionist	Miles traveled multiplied by mileage reimbursement rate divided by sample size	$69.39 (46.88)
Labor Cost of Travel	Travel to and from homes of study participants by interventionist	Wage rate + fringe benefit costs of interventionist multiplied by time spent in travel divided by sample size	$141.64 (127.16)
D. Total Cost of Travel (Mileage Reimbursement + Labor Cost of Travel)	Total travel costs	Sum of mileage reimbursement and labor cost of travel	**$211.03**
**Supervision Cost**			
Supervision of Screeners^~^	Supervision of screeners by trained senior center staff member	Wage rate + fringe benefit costs of supervisor and screener multiplied by time spent supervising employee then divided by sample size	$4.19 (n/a)
Supervision of Interventionists^&^	Supervision of interventionist by social work administrative staff of senior center	Wage rate + fringe benefit costs of supervisor and interventionist multiplied by time spent supervising employee then divided by sample size	$22.33 (n/a)
E. Total Cost of Supervision (Supervision by IHSP + Supervision of Interventionist)	Total supervision cost including supervisor reviewing, meeting and managing screener, meeting and managing interventionist, and managing care mangers and service staff by senior center	Sum or supervision of screeners and supervision of interventionist	**$26.52 (n/a)**
**Training^#^**			
F. Training	Training of senior center screeners and of interventionist to conduct intervention	Wage rate + fringe benefit costs of screeners and trainers, multiplied by time spent in training divided by sample size	**$0.9 (n/a)**
**Materials**			
G. Materials	All materials used by screeners and interventionists	Cost of materials used for screening and during the intervention divided by sample size	**$61.93 (n/a)**
**Adverse Events**			
H. Adverse Events	Cost of adverse events	Supervisor wage rate + fringe benefit costs, multiplied by time spent dealing with adverse events divided by sample size	**$0.32 (1.09)**
**Total Cost of BTB Per Person (A+B+C+D+E+F+G+H)**	Total cost of delivering BTB (screening plus 4 month intervention) per participant	Sum of sub-cost categories	**$584.64**
	Total cost of delivering BTB intervention per participant, per month		**$146.16**

* Represents: time spent delivering the intervention over the phone, in the home, or on site at the senior center

+ Represents: calls, visits, mailings, fax, preparation, documentation, see table below for detailed breakdown of costs

^~ ^In the table, cost is presented on a per participant basis, supervision costs were not linked at the participant level and thus standard deviations are not available. Cost of supervising screeners per supervisor (1) = $511.27 ($n/a). Cost of supervising screeners per screener (n = 17 screeners) = $34.97 (SD = 3.79).

^&^In the table, cost is presented on a per participant basis, supervision costs was not linked at the participant level and thus standard deviations are not available. Cost of supervising interventionist per interventionist (5) = 544.78 ($278.89).

^#^Cost to train 4 interventionists = $117

Time costs are monetized by multiplying time required for each task for each participant, times the wage rate plus fringe benefit costs of employees performing the tasks. Wage rates and fringe benefit costs (healthcare, disability, life insurance) are obtained directly from employers (senior center and university). Non-time related costs (program materials and mileage) are also recorded. Material costs include study documentation forms and education print materials provided to BTB participants. Interventionist travel expenses to and from participants' homes are captured per visit, and monetized based on reimbursement at the current government rate ($0.55/mile).

The average screening cost is $2.63 per participant; total cost of BTB (screening plus home intervention) is $584.64 over 4 months, or $146.16 per participant, per month. Time required delivering BTB at home ($197.31) and labor and mileage costs associated with travel to homes ($211.03) were the major contributors to total model costs. Total cost of BTB compares favorably to brand name antidepressants such as Paxil (paroxetine), Zoloft (sertraline), or Cymbalta (duloxetine) which cost approximately $100-$300 for a one-month supply [30]. Cost-effectiveness analyses of BTB from a societal perspective are currently underway.

## Discussion

BTB is designed to improve access to mental health services for older African Americans with depressive symptoms. This group represents a complex population as they frequently suffer multiple chronic conditions which place them at elevated depression risk, and have unmet care management needs, financial and housing constraints and limited access to mental health service options. Although clinical trials conducted in real world settings involve complex strategies, decisions and tradeoffs, interventions tested in the setting intended for its use may have greater potential for rapid implementation and sustainability if proven efficacious and cost effective.

Rapid completion of recruitment and successful enrollment, and high rate of detection of depressive symptoms among IHSP and community members, suggest that BTB resonates with the intended population. As screening for depressive symptoms and delivery of the home intervention has become normalized in senor center daily operations as a consequence of this trial, it appears feasible to integrate BTB in that setting and sustain at least the screening phase. An added benefit of embedding the trial in the senior center is that it results in capacity building such that staff can continue to conduct depression detection after the research is completed. Because intervention components are delivered in homes, BTB may appear resource-intensive. However, direct costs suggest otherwise and compare more than favorably to brand name antidepressants. Thus, feasibility of BTB as a practical trial embedded in a community-based aging service center is demonstrated in these important ways; success in training staff in depression screening, response by the community to recruitment efforts, enrollment of sample in 2 1/2 year period, low cost of screening and delivery of the intervention.

Although enrollment is closed, trial procedures are in progress. If BTB is efficacious and cost-effective, it will represent a promising new approach for depression detection and treatment for older African Americans warranting replication and implementation in other senior centers and the aging network in the USA. As context was carefully considered in constructing and testing BTB, its implementation and scalability potential may be enhanced. The participating senior center and research partner could serve as an implementation team to train others nationally in the model for rapid program expansion.

In conclusion, BTB was designed specifically for older African Americans. However, the intervention components and principles in which they are based (e.g., client-centered, tailoring, behavioral activation) have high relevance for other minority populations as well as developing similar symptom management and prevention programs for populations who may be hard-to-reach and remain undertreated for chronic illness and depression worldwide. Also, as a practical trial embedded in a service/practice setting, it is designed to build capacity in a community organization to engage in depression detection and treatment. The lessons learned from this collaborative academic-community partnership can help inform developing needed behavioral interventions for diverse older populations.

## Abbreviations

(BTB): Beat the Blues; (HIDEAS; Identifying Depression, Empowering Activities for Seniors): Healthy IDEAS; (PHQ-9): Patient Health Questionnaire; (IRB): Institutional Review Board; (QALY): Quality adjusted life year; (EQ5D): Euro-Quality of life; (HUI 2 and HUI3): Health utility measures; (USA): United States of America.

## Competing interests

The authors declare that they have no competing interests.

## Authors' contributions

LNG, principal investigator, developed study concept and design, developed research questions, developed intervention and model, oversaw scientific integrity and interpretation of data, and assumed primary conceptual development and writing responsibility of manuscript. LFH, co-investigator, helped develop intervention and model, oversaw implementation of model at senior center site, assisted in description of senior center activities and intervention description table, and provided a critical review and editing of manuscript. MM, project manager at senior center site, provided supervision and oversight of senior center screeners and interventionists, assisted in preparation of intervention description and table, and provided a critical review and editing of manuscript. NLC, project manager at research site, provided supervision of research interviewers and data collection by interviewers and interventionists, conducted literature searches and provided a critical review and editing of manuscript. EJ, project coordinator for the cost analyses, helped to coordinate collection of cost data, conducted cost analyses, conducted literature searches relevant to cost analyses, prepared cost table for manuscript and provided a critical review and editing of manuscript. LTP, co-investigator of cost analysis study of BTB model, assisted in cost analyses, interpretation of cost data and write up cost description and provided a critical review and editing of manuscript. All authors read and approved the final manuscript.

## Pre-publication history

The pre-publication history for this paper can be accessed here:

http://www.biomedcentral.com/1471-2318/12/4/prepub
